# Assessment of ultraviolet radiation impact on human skin tissue using double-exposure digital holographic interferometry

**DOI:** 10.1117/1.JBO.30.2.025001

**Published:** 2025-02-10

**Authors:** Gloria Frausto-Rea, María del Socorro Hernández-Montes, Fernando Mendoza Santoyo, Noé Alcala Ochoa, Edgard Efrén Lozada Hernández

**Affiliations:** a Centro de Investigaciones en Óptica, León, México; b Hospital Regional de Alta Especialidad del Bajío and Instituto Mexicano del seguro social para el bienestar, León, México

**Keywords:** ultraviolet radiation, human skin tissue, digital holographic interferometry, mechanical response

## Abstract

**Significance:**

We are all exposed to ultraviolet (UV) radiation coming from the Sun, electronic devices, and artificial sources used in medicine, industry, cosmetics, and other fields, and as it can penetrate the skin, it poses a health risk. In this research, the effects of UV radiation on human skin exposed to different energy doses are evaluated using digital holographic interferometry (DHI), which is proposed as a useful tool to assess the changes caused by skin surface displacement and stiffness values. These two indicators, and their representation in pseudo-three-dimensional (3D) images, will be used as biomarkers, and their quantification will help to better understand the effects of UV rays on human skin.

**Aim:**

This research is centered on studying human skin tissue samples (HSTs) with double-exposure DHI; this non-invasive optical technique is able to detect alterations in its mechanical response as it changes caused by UV radiation falling on the skin surface, and such response is compared with the one of non-irradiated samples allowing us to correlate the changes in displacement and stiffness resulting from exposure to different doses of UV radiation.

**Approach:**

Acoustic waves are sent to the HST to induce vibrations and displacements on their surface; the resulting vibration patterns are monitored through an out-of-plane sensitive DHI setup. The full-field-of-view quantification of the displacements in the z-direction (normal to the surface) is quickly determined by processing the digital holograms, and with the amplitude of the displacements, skin stiffness is calculated. Both the surface displacements and their corresponding stiffness values correctly reveal the effects caused by the different UV radiation doses falling on the HST surface, a matter discussed in detail.

**Results:**

The resonant frequencies and the 3D shape of the vibration showing the displacement and stiffness of human skin with and without radiation were found, and graphs were constructed using those data. A negative correlation is observed between the amount of UV energy applied and the changes in displacements, whereas a positive correlation is observed between stiffness and UV dose. The plot serves as a calibration plot and thus can be used to predict, from the optical data, the displacement and stiffness as a function of the UV dose. In addition, some critical changes in skin stiffness may indicate aging or dehydration in the skin, and this may be useful to achieve better skin care. These data indicate that UV light induces skin stiffening. The amplitude variation in displacement/strain and stiffness allows differentiation between skin tissues without and with UV radiation.

**Conclusions:**

The optical non-invasive DHI technique offers a whole field of view assessment of the UV effects on the HST without touching the skin. With the aid of DHI, it is possible to retrieve the pseudo-3D phase map of the skin, and from it, valuable data on the displacement of the skin surface, and thus, its stiffness can be obtained; in addition, many potential benefits can be derived from its use, such as protection against skin diseases.

## 1 Introduction

The penetration depth of ultraviolet (UV) light into the tissues of the human body is low; so, the biological effects are mainly limited to unprotected skin and eyes.^1^^–^^3^ The skin is the fine tissue layer that constitutes the body’s natural integumentary system, serving as a barrier to shield the body from external and internal conditions that may trigger unwanted biological reactions. UV radiation has increased over the years, causing irreversible damage to the human skin, i.e., the skin becomes fragile resulting in changes in its biomechanical properties, hence the importance of assessing the effects on its elasticity.^4^ It is recognized that low-level exposure to certain wavelengths of UV light provides some health benefits, as in the case of the synthesis of vitamin D3, which promotes calcium absorption in the body, and good levels of vitamin D are related to reducing the risk factor for the development of different types of cancer among others.^5^^,^^6^ In contrast, overexposure to UV light can result in some adverse health effects, such as erythema, photoconjunctivitis, and photokeratitis in the short term, as well as premature skin aging and skin cancer as a result of frequent long-term exposure.^7^^–^^12^ Prolonged exposure to UV rays can cause microdamage to the skin’s elastic fibers, leading to a denser structure with diminished elasticity. Chronic exposure to UV radiation can cause degenerative changes in cells, fibrous tissues, and blood vessels, which over a lifetime can lead to non-melanoma skin cancer.^13^ These internal degenerative changes are reflected on the skin surface. Therefore, conducting specific mechanical measurements on the surface of biological samples using non-invasive optical methods has proven useful in generating new data to characterize various sample conditions.

Numerous techniques have been devised for assessing skin stiffness, including optical elastography, fluorescence-based optical elastography (FOE), optical coherence elastography, and photoacoustic elastography (PAE), among others. These methods have demonstrated their utility in detecting and monitoring a range of diseases by evaluating mechanical characteristics. Also, these and other optical methods have the value of being non-invasive, high image resolution, and the ability to observe changes in the internal structure of the tissue such as when using optical coherence tomography (OCT) and providing three-dimensional (3D) images, but others need fluorescent markers such as FOE or may be limited to certain tissues and have limited penetration as in the case of OCT and PAE.^14^^–^^16^ In contrast, digital holographic interferometry (DHI) is an optical non-invasive technique employed in the analysis of diverse objects, including those with intricate surfaces, due to its capability to measure deformations and mechanical characteristics precisely and non-invasively. The high resolution, down to nanometers, achieved with DHI positions it as a favorable option for examining subtle, imperceptible alterations. DHI facilitates the examination and monitoring of the entire surface of soft tissues allowing the characterization of stiffness, optical, and biomechanical properties. In addition, it is a method that yields pseudo-3D imagery.

Digital holographic interferometry is a non-destructive (non-invasive) technique belonging to the discipline of optical metrology, which is used to inspect and evaluate materials, components, or structures without causing permanent damage or altering their integrity, and serves to obtain detailed information about the optical and biomechanical properties of the object. DHI is based on interferometry and, thus, can obtain displacement measurements of objects subjected to many types of stimuli with a sensitivity below the wavelength of the light used for the measurements; in addition, the technique offers full field of view of the objects under inspection and is quantitatively accurate–precise and robust.^17^^,^^18^ Through this technique, it is possible to create three-dimensional images that provide a vision of depth and realism. Studies have been performed when the sample is static or dynamic, the latter happening through the application of sound, and being due to sound-induced motion in different types of objects, which causes their masses to vibrate or oscillate. For example, natural phenomena (such as earthquakes and tornadoes), as well as some vital human functions (such as the heartbeat, hearing, seeing, speaking, walking, or breathing), involve vibrations.^19^^–^^23^ The amplitude, frequency, resonance, and periodicity of the vibrations can be associated with the good or bad performance of the samples, and so, any failure/defect could be related to vibrations because these can cause wear and tear or malfunction of vital organs. Therefore, studying the vibrations will provide crucial information about the functioning of the biological samples to be investigated.^24^^,^^25^

The literature reports indicate that infections and diseases in biological samples may result in altered mechanical properties of the tissue. These changes can serve as indicators to distinguish between affected samples and controls, directly correlating with the tissue’s condition.^26^^,^^27^ The DHI technique is promising for medical and biological applications, such as the study of tissues, cells, and others, due to the unique properties of the technique, namely, its non-invasiveness and full-field imaging capability.^28^^,^^29^

In this work, we study normal (non-UV-irradiated) and UV-irradiated human skin tissue (HST) using double-exposure DHI to non-invasively measure, in a full-field of view, their unique vibration patterns, with their corresponding surface deformation and their stiffness. The HSTs are subjected to acoustic excitations on their backside (not seen by the camera sensor used in DHI), which propagate through the skin and cause the observable sample surface (seen by the camera sensor) to vibrate. Our findings suggest that the monitoring of distinctive vibration patterns, displacement amplitude, and stiffness may provide a feasible method of determining UV skin affectation. The results show amplitude variation in displacement/deformation and stiffness, allowing differentiation between skin tissues without and with UV radiation. This approach is expected to contribute to a better understanding of UV effects on HST stiffness as a function of applied doses, and it holds significance in the field of biomedical optics and even in mechanical areas. The reliability and robustness of the proposed approach are first tested on a known aluminum sample, a phantom skin, and skin samples under different UV doses. Ten different doses were applied to the HST, and their comparison is shown in Sec. 3.

## 2 Materials and Method

### 2.1 Double-Exposure Digital Holographic Interferometry

**Fig. 1:**
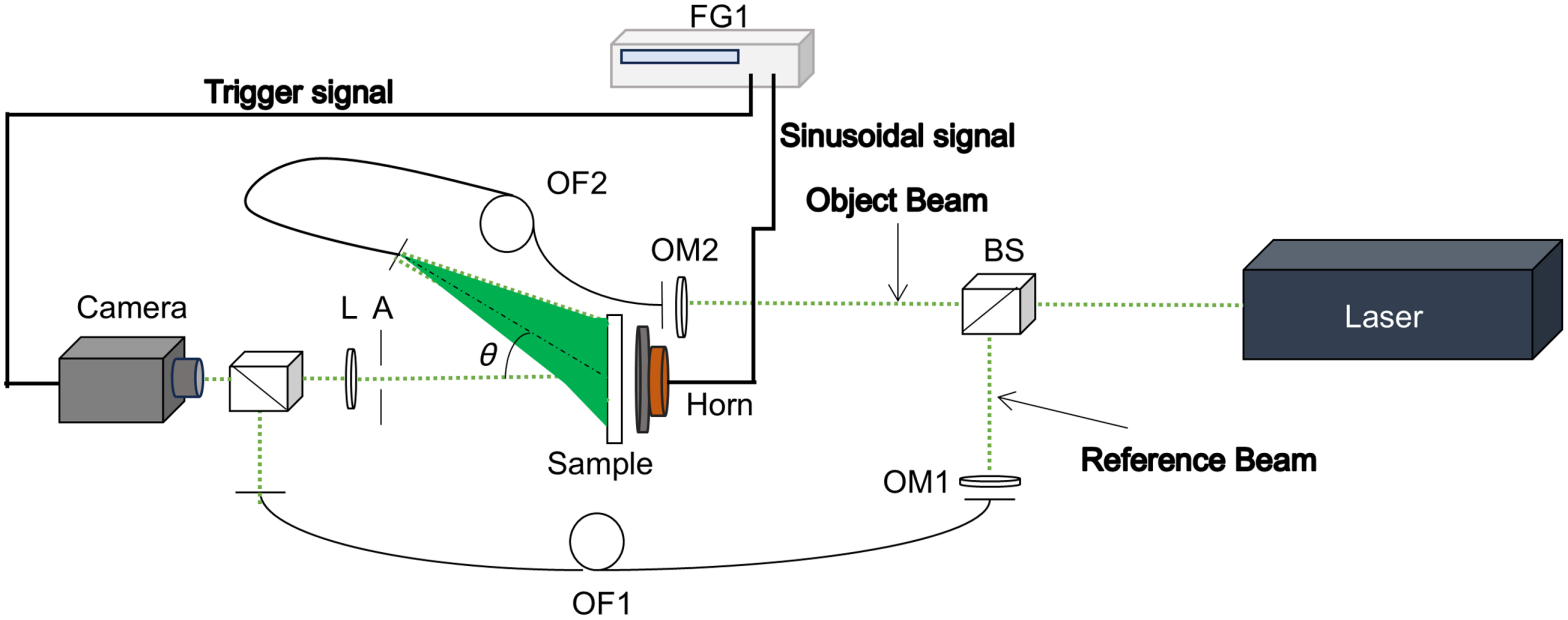
Optical configuration with sensitivity in the z-direction.

Digital holographic interferometry requires simultaneously recording a reference and an object beam on the camera sensor to create a digital hologram; it is also a two-stage technique where two object scenes are recorded, one for the object at a known position and the second for the object at any other position (refer to Fig. 1), where an out-of-plane sensitive setup is drawn. The created interference is recorded on the camera sensor, and it can be expressed as

$$I(x,y)=a(x,y)+c(x,y)+c^{*}(x,y).$$ (1)

The sign * denotes the complex conjugate term, a(x,y) is the sum of the reference and object beam intensities and may be considered as a constant, and $c(x,y)=\frac{1}{2}b(x,y)e^{i\varphi (x,y)}$ where b(x,y) is the modulation factor. φ is the phase of the fringe pattern, and (x,y) denotes the two-dimensional (2D) pixel coordinates on the camera sensor. The resulting pattern is parsed and used to extract information about the object’s properties.

As a first step, we proceed to identify the unique vibration fringe patterns of the object by placing an acoustic sound on its back side. For this study, it is vital to acquire two hologram images (double-exposure method) with the charge-coupled device (CCD) camera: one before ($I_{R}$) and the other ($I_{o}$) after introducing the vibration to the object under study. $I_{R}$ is stored as the reference hologram and then subtracted from $I_{o}$ to obtain the vibration pattern and display the intensity modulation ($I_{m}$) as

$$I_{m}=|I_{o}-I_{R}|.$$ (2)

After identifying the vibration patterns, digital processing of the patterns $I_{o}$ and $I_{R}$ is performed to recover the individual phases $\varphi_{o}(x,y)$ and $\varphi_{R}(x,y)$, respectively. It must be remembered that the magnitude displacements on a micrometer scale are contained in its optical phase. The recovery of the optical phase difference $\Delta \varphi =\varphi_{o}-\varphi_{R}$ is achieved using the spatial carrier method that involves the Fourier transform^30^ because the off-axis DHI introduces the spatial carrier by tilting the reference beam at the beam combiner cube (BC) in the optical setup (Fig. 1).

Subsequently, the 2D Fourier transform algorithm is applied to $I_{o}$ and $I_{R}$ expressed with Eq. (1), resulting in^31^

$$FT\{I(x,y)\}=A(u,v)+C(u-f,v)+C^{*}(u+f,v).$$ (3)

In Eq. (3), A is the low-frequency background illumination amplitude, and (u,v) are the coordinates in the Fourier frequency domain; C(u−f,v) and $\;C^{*}(u+f,v)$ represent, correspondingly, the ±1 (lobe) of the resulting image spectrum. It is necessary to maintain only one spectral lobe to recover the optical phase; so, a band-pass filter is applied to Eq. (3) to remove the complex conjugate and A term. The filtered lobe is moved to the center of the frequency coordinates, and after applying the inverse Fourier transform, a complex number is obtained, from which the phase information is recovered. Thus, we will denote with $I_{\mathrm{OIT}}$ and $I_{\mathrm{RIT}}$ the respective complex images obtained after applying this filtering process to $I_{o}$ and $I_{R}$, which let us calculate the phase difference Δφ with the following equation:

Δφ(x,y)=arctan[Re(IRIT)Im(IOIT)−Im(IRIT)Re(IOIT)Im(IRIT)Im(HIOIT)+Re(IRIT)Re(IOIT)], (4)

where Re and Im denote the real and imaginary parts of the complex numbers, respectively. The phase Δφ is recovered in the form of a wrapped phase map restricted to the interval −π,π]; so, it is necessary to unwrap it to obtain a smooth optical phase denoted by $\Delta \varphi^{\prime }$, which is used to get the displacement map (w), by

$$w(x,y)=\frac{\lambda }{2\pi (1+\cos\;\theta )}\Delta \varphi^{\prime },$$ (5)

where θ is the illumination angle (between the observation and illumination directions), and λ is the wavelength of the illumination source used in the optical system.

The surface displacement field maps are used to compute the stiffness, a parameter that will help us to evaluate how UV radiation affects/injures the sample in its flexibility, i.e., higher rigidity is an indicator that the sample has lost flexibility or elasticity.^32^^–^^36^ Thus, the loss of elasticity in this study is a bio-marker of skin aging due to the application of various doses of UV radiation. Stiffness (k) is evaluated using the following equation:

$$\;k=\frac{F}{w},$$ (6)

where F represents the force applied to the object under analysis, and w refers to the resulting deformation. To find the force, first, sound intensity ($L_{p}$) is measured in decibels sound pressure level using an HER-402 digital sound meter, which was positioned 5.5 cm away from the loudspeaker. Pressure ($P_{r}$) is determined using the expression $L_{p}=20\;\log(\frac{P_{r}}{P_{\mathrm{ref}}})$, where $P_{\mathrm{ref}}$ is the reference pressure, and its value is 20  μPa.^37^^–^^39^

With this information, and knowing the object’s area (A) in meters, it is possible to get the force (F) through

$$F=P_{r}A.$$ (7)

### 2.2 Experimental Layout

Figure 1 shows an out-of-plane digital holographic interferometry setup used to analyze biological and mechanical samples. The optical setup consists of a Cobolt Samba green laser at 532 nm and 2-W peak power. The laser beam is directed to a beam splitter (BS), where it is split into a reference and an object beam; both beams are sent to two microscope objectives (OM1 and OM2) to couple the light into a single mode optical fiber (OF1 and OF2, respectively). The object beam is responsible for illuminating the sample, and the light backscattered by the object is collected by a system comprising a lens (L) with an aperture (A), then directed to the camera sensor by a BC, and overlapped with the reference beam to create the digital hologram. The camera used is a PCO.pixelfly, which can be set to a resolution of either 1392×1040 or 800×600  pixels, with a pixel size of 6.45  μm.

A hologram capture synchronization process was implemented to ensure that the recording of the holograms coincides with the maximum peak of the sinusoidal sound signal to quantify the displacement of the sample surface at this point. For this purpose, a function generator (FG1) model AFG3022C was used, which has two output channels configured as follows: channel 1 provides a pulse waveform, and it was used as a trigger signal that goes to the input of the CCD camera to start and control the acquisition and recording of the holograms, whereas channel 2 provides a sinusoidal signal that feeds the horn to produce a sound signal to excite the sample. Both channels were activated simultaneously, and to ensure the synchronization of the signals, they were visualized through a digital oscilloscope.

**Fig. 2:**
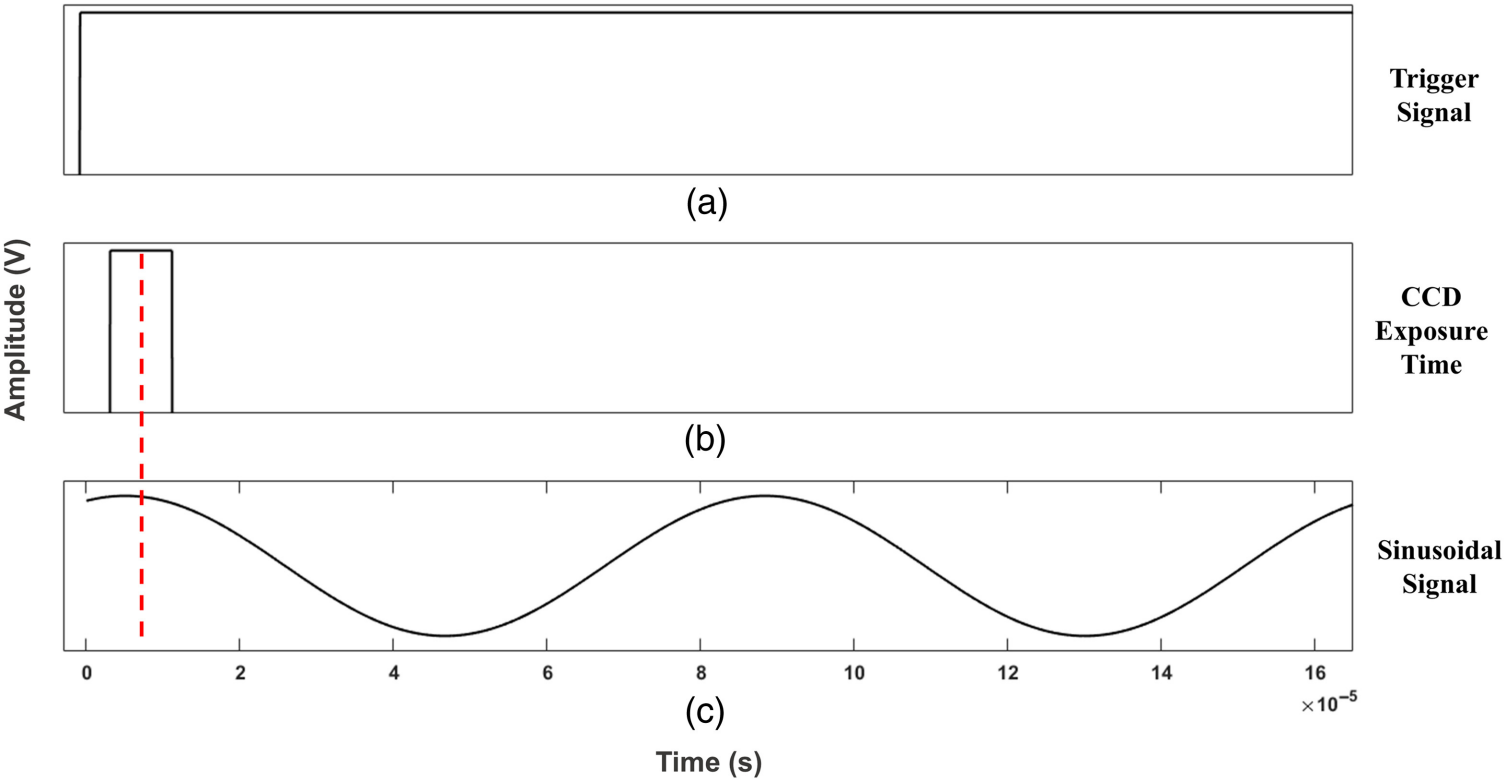
(a)–(c) Signals used for the synchronization stage. The red dashed line indicates where the images were captured.

Figure 2 shows the waveform diagram signals: panel (a) shows the trigger signal, panel (b) is the exposure signal, and panel (c) is the sinusoidal signal that is used to activate the horn.

### 2.3 Samples

**Fig. 3:**
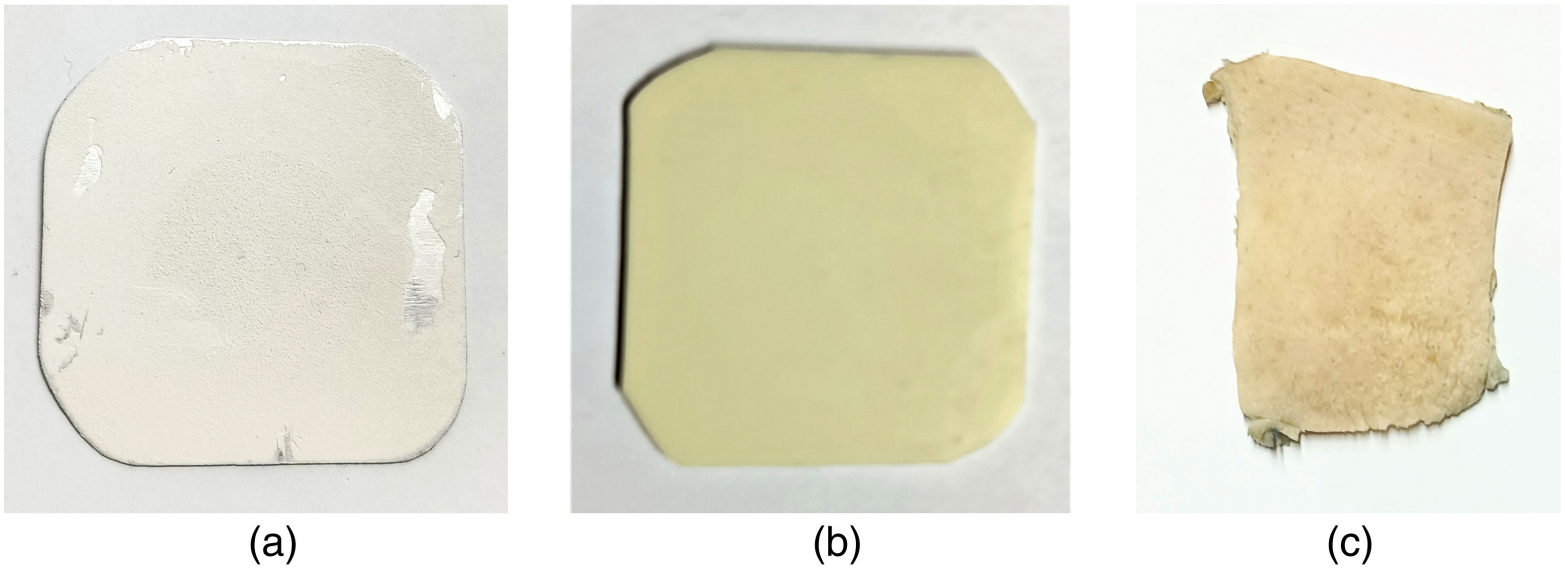
Specimens used for the study. (a) Metal plate. (b) Skin phantom. (c) Human skin.

Figure 3 shows the specimens used in this study: metal plate, skin phantom, and human skin, the latter being the sample of interest in this study.

#### 2.3.1 Metal plate

To verify the correct performance of the experimental setup and methodology, a thin circular plate made of aluminum with a diameter of 20 mm and a thickness of 3.81 mm was first evaluated. The plate was held at its edges by a mechanical mount and was investigated with the DHI layout shown in Fig. 1. The surface of the aluminum plate was sprayed with a light coat of white paint to obtain more backscattered light from its surface. The natural resonant frequencies of the plate were identified, and the vibration patterns and the corresponding displacement of the plate were found.

#### 2.3.2 Skin phantom

An additional study was carried out with a material made of silicone similar to skin tissue. The skin phantom has a thickness and diameter of 1 and 20 mm, respectively. The phantom was secured at its edges by mechanical mount and examined using the DHI setup depicted in Fig. 1. The natural resonance frequencies were identified, along with the vibration patterns, as well as the corresponding phantom displacements and stiffness. After confirming that the system functioned correctly with the plate and mimic skin as samples, we proceeded to conduct the study using HST, which is the sample under investigation in this research.

#### 2.3.3 Skin tissue preparation

Sections of fresh skin tissue were obtained from a patient undergoing abdominoplasty procedures in the plastic surgery clinic of the High Specialty Regional Hospital located in the city of León, Guanajuato, Mexico. It is important to mention that the patient was not subjected to any risky procedure different from what the subject was programmed for and that the rest of the skin, once the sample was obtained, was taken to the pathology service where it was submitted to the evaluation (for disposal). A study protocol approved by the hospital board was strictly followed, and the participating patient signed a consent form before surgery. This study was approved by the hospital’s ethics and research committee with registration number: CI/HRAEB/0008/2023. The hospital did provide sections of skin tissue from the patient between the ages of 27 and 50. Each skin section was stored in a formaldehyde solution and subsequently refrigerated at ∼2°C immediately after surgery. A sample of ∼6×6  cm in size was prepared by removing hair from the skin surface with scissors, and the subcutaneous adipose tissue with a razor blade, and then kept in 0.9% saline; care was taken to preserve the stratum corneum layer of the epidermis of the skin. During the experiment, the sample was thawed by immersion in saline solution to prevent dehydration of the sample.

## 3 Experimental Results and Discussion

### 3.1 Metal Plate

The thin circular aluminum plate was tightly held in place (i.e., clamped around its entire circumference) through the use of a mechanical circular mount. A loudspeaker was used to excite the plate with sound (see Fig. 1). A frequency sweep was performed to determine the resonant frequencies and their corresponding vibration patterns.

**Fig. 4:**
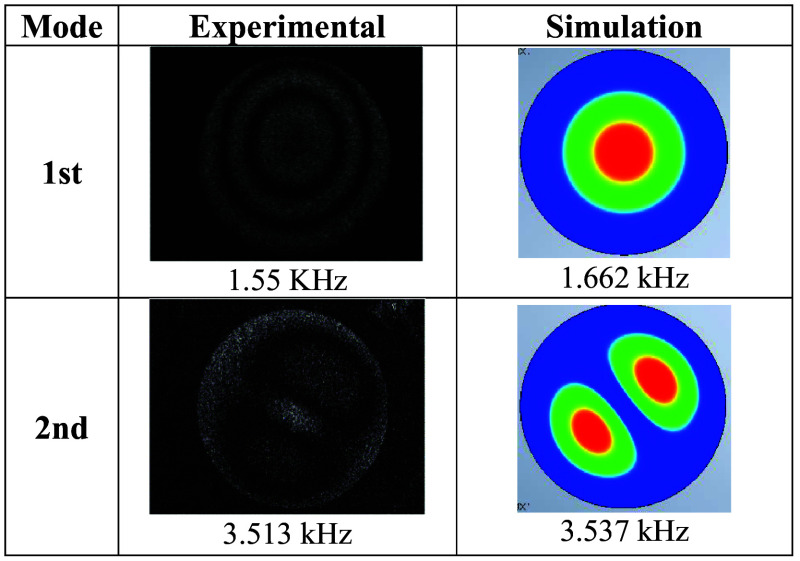
Mode shapes and frequency results of the circular aluminum plate.

First, a reference hologram $I_{R}$, of the sample was taken without sound; then, the sample was subjected to sound waves, creating a modified-object hologram $I_{o}$; Eq. (2) was used to compute the holograms that resulted in the characteristic vibration fringe patterns. Figure 4 shows the experimental and computationally simulated results obtained for the first and second resonant modes, with both methods showing a very good match between experimentation and theory. The simulation was made with the Computer-Aided Design (CAD) software, using the circular plate designed with the characteristics described in Sec. 2.3.1, as the model, and with the boundary conditions being that the plate be clamped around its entire circumference. The similarity between the experiment and the simulation was calculated, finding a value of 93.26% for the first mode and 99.3% for the second mode; notice the slight shift in frequency values, a feature due to the CAD software used.

Once the resonant vibration patterns were identified, the first mode at 1.55 kHz was selected to quantify the displacements. The holograms were processed following the steps indicated in Secs. 2.1 and 2.2, and their displacement maps were determined using Eq. (5). The recording of the hologram by the camera was synchronized with the sound wave at its maximum level, as described in Sec. 2.2. For the frequency of 1.55 kHz, the pulse waveform used as a trigger signal was set with an amplitude of 4 Vpp, a duty cycle of 99.993%, and a period of 1.36 s; the sinusoidal sound signal was configured with an amplitude of 5 Vpp (delay phase = 75.72 deg). With these parameters, the holograms were captured at the maximum peak of the sound wave.

**Fig. 5:**
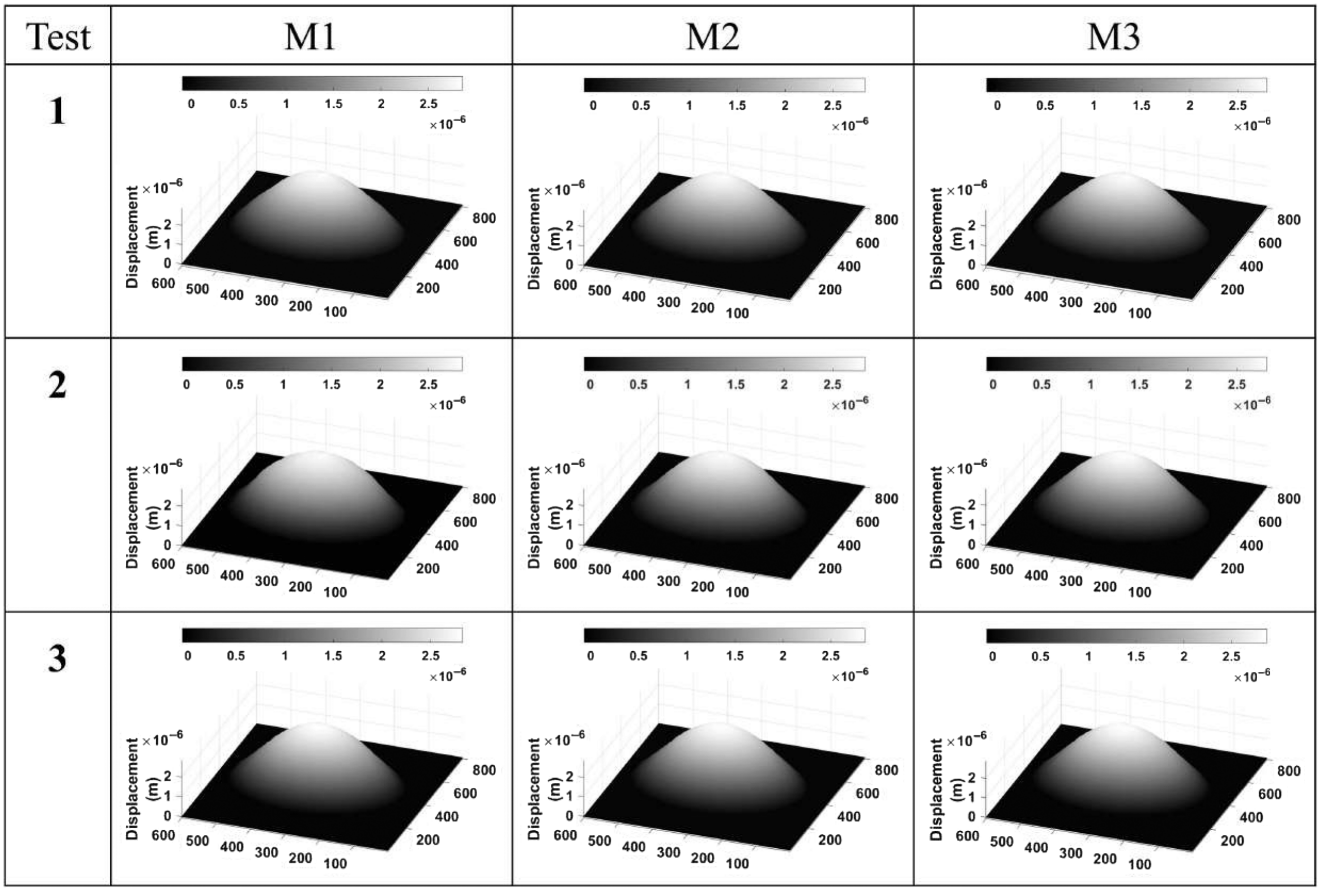
Representative displacement maps taken at 1.55 kHz of aluminum plate, showing the repetitiveness of the measurements.

Figure 5 shows a selected group of three test measurements of the circular plate displacement maps (M1, M2, and M3). It is worth mentioning that a minimum of 10 tests were carried out to verify that the synchronization process was reliable, as well as to evaluate the repeatability of the measured displacements. According to the results shown in Fig. 5, it can be visualized that the values of the displacement for each of the tests and between each series are quite similar. The average for the maximum displacement was ∼2.84  μm.

With these accurate and reliable results obtained from the plate, we moved on to evaluate human skin tissue.

### 3.2 Skin Phantom Study

**Fig. 6:**
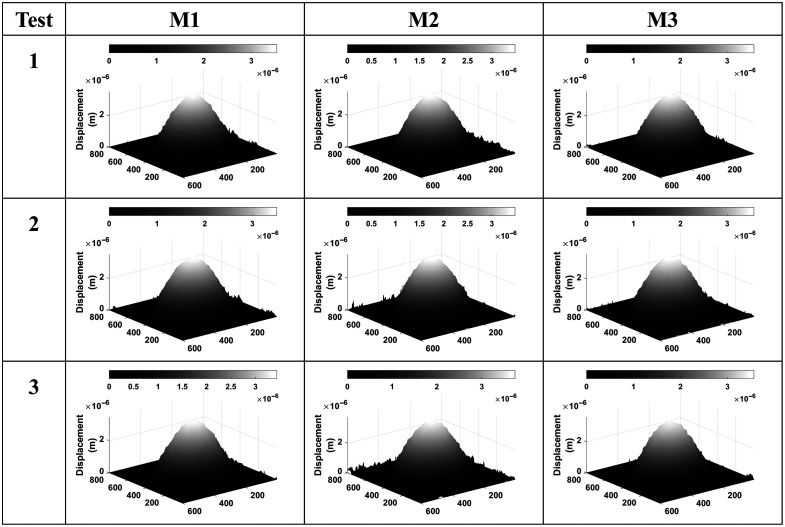
Representative displacement maps taken at 370 Hz, showing the repetitiveness of the measurements. It is observed that the displacement was higher for the skin phantom.

**Fig. 7:**
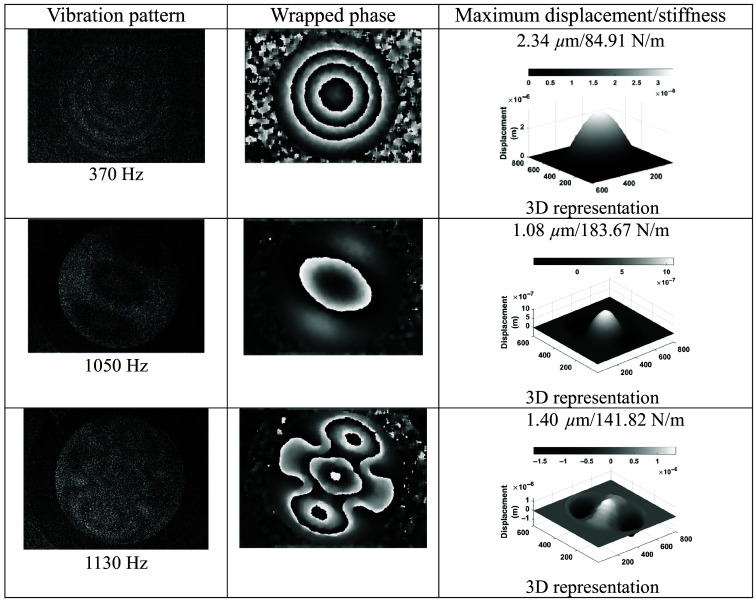
Skin phantom results at different stimuli frequencies showing its elasticity.

The skin phantom was excited in the frequency range from 300 to 2000 Hz, identifying the fundamental natural frequencies of resonance with the first mode located at 370 Hz. Using this frequency for demonstration purposes, Fig. 6 displays the phase maps and displacement patterns derived from the quantitative analysis of the 3D phantom measurements. Multiple tests were conducted to ensure the consistency of the results. Three images were chosen for three separate tests to observe the sample’s behavior. The material showed a higher propensity for movement because it responded to a higher number of sound frequencies, a feature attributed to its flexibility, see Fig. 7. The averages for maximum displacement and stiffness recorded were 2.34  μm and 84.91  N/m, respectively.

### 3.3 Skin Tissue Results and Discussion

A human skin tissue sample, with a thickness of 2 mm, a diameter of 20 mm, and a surface area of $∼0.000314\;m^{2}$, was analyzed to obtain its vibration patterns, the displacement field maps, and the stiffness before and after UV radiation. The skin studied was held in place by a mechanical circular support and subjected to a sound wave pressure of 0.6325 Pa. With these data, the applied force was calculated using Eq. (7), resulting in 198.705  μN.

#### 3.3.1 Skin tissue without UV radiation

**Fig. 8:**
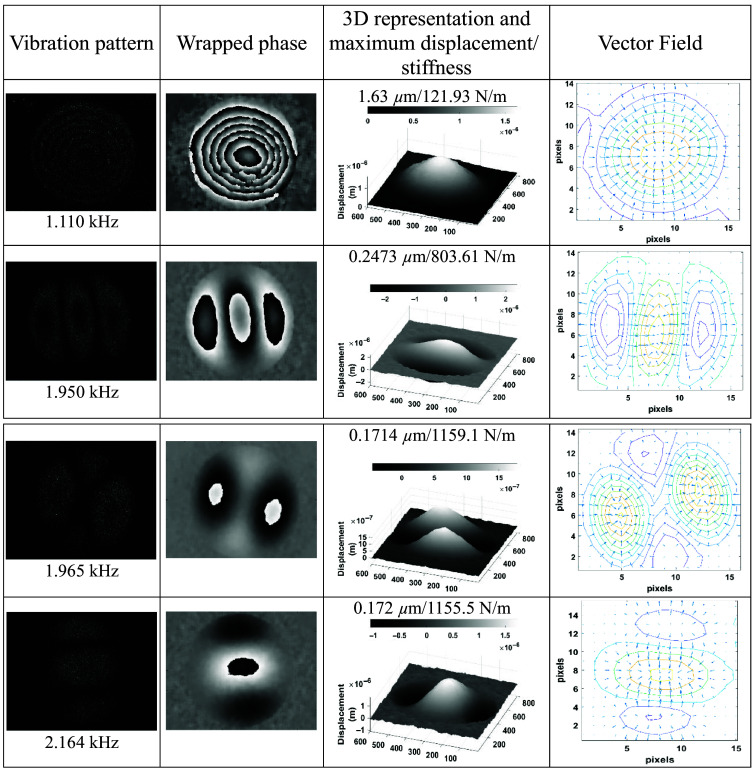
HST results without UV radiation showing its elasticity.

To begin, the tissue was studied without UV radiation in the optical setup shown in Fig. 1, a configuration used to identify the response frequencies and vibration patterns. Figure 8 shows the vibrating patterns obtained by correlating two holograms using Eq. (2), the resonant frequencies found, the wrapped optical phase obtained with Eq. (4), the 3D shape of the vibration surface showing displacement computed by Eq. (5), the stiffness data obtained using Eq. (6), and the vector field maps. The latter represents the direction of change of the deformation generated in the sample in the form of 2D scattered vector fields, obtained using the gradient function $\left\langle w_{x}(x,y),w_{y}(x,y)\right\rangle$. It is observed that the skin is very susceptible to vibrations over a range of frequencies, this being indicative of the flexibility of the skin without UV radiation.

After finding the vibration patterns, we moved on to quantifying the displacements and the elasticity of the skin sample; the first mode found at 1.11 kHz was selected to perform the study. The sample without radiation was taken as a control sample, and the tests were performed under the same experimental conditions. The reference and modified holograms were recorded by the camera, and the capturing process was synchronized with the external trigger and with the sound wave at its maximum level, as was described in Sec. 2.2. The synchronization signals were configured as follows: the sinusoidal wave was set at 1.11 KHz, with an amplitude of 4 Vpp and a phase of 83.77 deg, and the pulse trigger signal had a period of 1.37027 s and an amplitude of 4 V to ensure that the holograms were recorded at the peak of the sound wave. The holograms were processed following the steps indicated in Sec. 2.2.

**Fig. 9:**
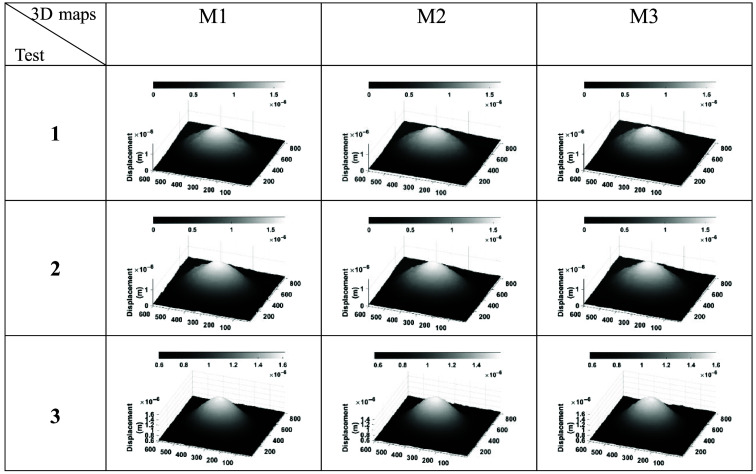
Displacement maps for three different tests in skin tissue without UV radiation taken at 1.11 kHz showing the repeatability of the measurements.

Figure 9 shows the displacement maps of the tissue without radiation; we only show three tests, picked at random, and each one displays three images as 3D maps. It is worth mentioning that a minimum of 10 tests, with 10 measurements each, were performed to verify the repeatability of the measured displacements. The results show that the displacement values for each of the tests and among series are quite similar. The mean displacement was 1.6475  μm, and the mean stiffness was 120.6825  N/m. The variance and standard deviation of the displacements/stiffness were calculated resulting in $0.0025\;{\mu m}^{2}/12.8685\;N^{2}/m^{2}$ and 0.0498  μm/3.5873  N/m, which correspond to percentage deviations of 3.02% and 2.97% correspondingly.

#### 3.3.2 Skin tissue with UV radiation

Next, we moved on to the skin exposed to near UV radiation. For this purpose, a UV irradiation chamber (model BS-02 UV) was used, and the control sample was irradiated with different doses of energy.

The radiated skin was inspected under the same experimental conditions with the setup displayed in Fig. 1, and the holograms were captured for the selected vibration mode at 1.11 kHz. The recording of the holograms was set with the same parameters of synchronization that were used in the sample without UV radiation. The processing used to analyze the two-state holograms was described in Sec. 2.1.

To determine the dose and exposure time required to irradiate the sample, we considered the minimal erythematic dose (MED) for lightly pigmented white skins, whose value ranks from 0 to $99\;J/m^{2}$ for a UV index of 1 to 1400 to $1499\;J/m^{2}$ for a UV index of 15 per hour.^40^ With the objective of producing affectation in the biological tissue, but taking care not to damage the integrity of the sample, a minimum value of $0.0030\;J/{\mathrm{cm}}^{2}$ was selected to be the initial dose to be applied to the HST; subsequently, increments of 0.003 were introduced, up to a final irradiance of $0.030\;J/{\mathrm{cm}}^{2}$. During each UV exposure, the sample was irradiated for 10 s.

**Fig. 10:**
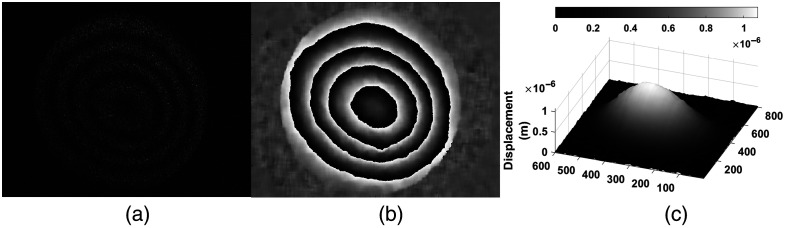
Skin tissue patterns after radiation at 1.11 KHz. (a) Fringe pattern. (b) Wrapped phase. (c) 3D map.

At first glance, no difference in the shape of the recorded vibration mode was observed; however, we can infer that irradiation decreased the skin moisture because the sample exhibited a stiffer and less elastic behavior with decreasing displacements. Figure 10(a) shows the fringe pattern, panel (b) is the wrapped phase, and panel (c) shows the displacement map. Comparing this figure with the first vibrational mode at 1.110 kHz in Fig. 8, before irradiation, a decrease in the maximum displacement with a value of 1.06  μm is observed due to skin exposure to UV radiation.

Table 1 presents the maximum displacement and stiffness results of the skin achieved with 10 different irradiance doses (first column) in the UV chamber. The second row shows the results obtained without radiation (W/R), taken as a control sample to compare the changes in the sample with UV radiation. The first vibration mode is set for all experimental tests and to analyze the behavior of the sample. The resulting displacement and stiffness values are used to assess the impact of UV radiation. Ten measurements were performed, from which we randomly picked three measurements (M1, M2, and M3) as an example.

**Table 1:** Displacement and stiffness results were taken at 1.11 kHz for 10 different radiation doses applied to skin tissue.

UV radiation ($J/{\mathrm{cm}}^{2}$)	M1	M2	M3
	Displacements/stiffness (μm)/(N/m)	Displacements/stiffness (μm)/(N/m)	Displacements/stiffness (μm)/(N/m)
0 (W/R)	1.64/121.93	1.63/121.28	1.60/124.41
0.003	1.53/129.62	1.51/131.82	1.51/131.92
0.006	1.48/134.34	1.38/143.77	1.37/144.87
0.009	1.43/138.51	1.33/149.26	1.35/146.78
0.012	1.40/142.13	1.31/151.60	1.25/161.05
0.015	1.32/150.86	1.30/151.67	1.23/161.18
0.018	1.28/155.41	1.27/156.50	1.22/162.48
0.021	1.26/158.31	1.25/158.95	1.17/169.14
0.024	1.06/187.21	0.96/206.48	0.85/233.17
0.027	0.95/208.95	0.85/233.64	0.84/237.05
0.030	0.73/272.82	0.68/292.67	0.67/297.14

The data in Table 1 indicate displacement variability due to changes in skin elasticity/rigidity. The maximum displacement is observed in the W/R sample, and it is noticed that when the UV dose is applied, the displacements decrease as the stiffness increases, causing its elasticity to change. Both parameters correctly reveal the UV effects which can be associated with the photoaging caused on the surface of HST for different radiation doses, as occurs when the skin is exposed to sun or other sources of UV radiation.

**Fig. 11:**
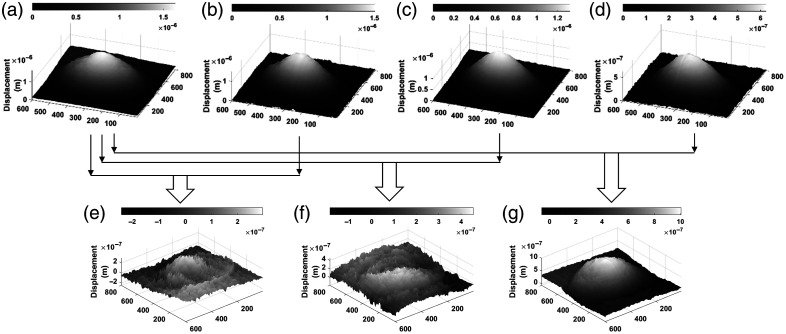
3D images taken at 1.11 KHz of the sound wave. (a) Without UV (control sample) and irradiated with (b) $0.003\;J/{\mathrm{cm}}^{2}$, (c) $0.015\;J/{\mathrm{cm}}^{2}$, and (d) $0.030\;J/{\mathrm{cm}}^{2}$. (e)–(g) Result of the subtraction of the displacements between the control sample and those in panels (b)–(d), respectively.

Figure 11 shows four displacement maps corresponding to different states of the test: without radiation, shown in Fig. 11(a), and tests with initial, intermediate, and final radiation values, shown in Figs. 11(b)–11(d), respectively. The dependence of the value of the displacements on the radiation dose, where a smaller displacement is obtained when the radiation is high and vice versa, is remarkable. The 3D displacement maps provided a quantitative measure at each of the points that make up the maps of the changes in the skin, and consequently, in its elasticity, due to exposure to UV radiation. With this information, it is possible to measure changes in skin properties that are not visible to the naked eye. These parameters are indicative of the skin’s affectation.

The lower part of Fig. 11 shows the evolution of the displacement, for which the image without radiation Fig. 11(a) is taken as a baseline and subtracted from each image with radiation Figs. 11(b)–11(d), resulting in the images shown in Figs. 11(e)–11(g), in that order. It is observed that for a low radiation dose, the tendency of the value of the displacement difference will be smaller, whereas with the increment in the radiation dose, it will be higher. In Fig. 11(d), the displacement map is more noticeable because the difference in displacement quantification is larger due to the fact that the sample was subjected to more radiation, and therefore, its elasticity was affected, producing a smaller displacement when the same sound force is applied.

**Fig. 12:**
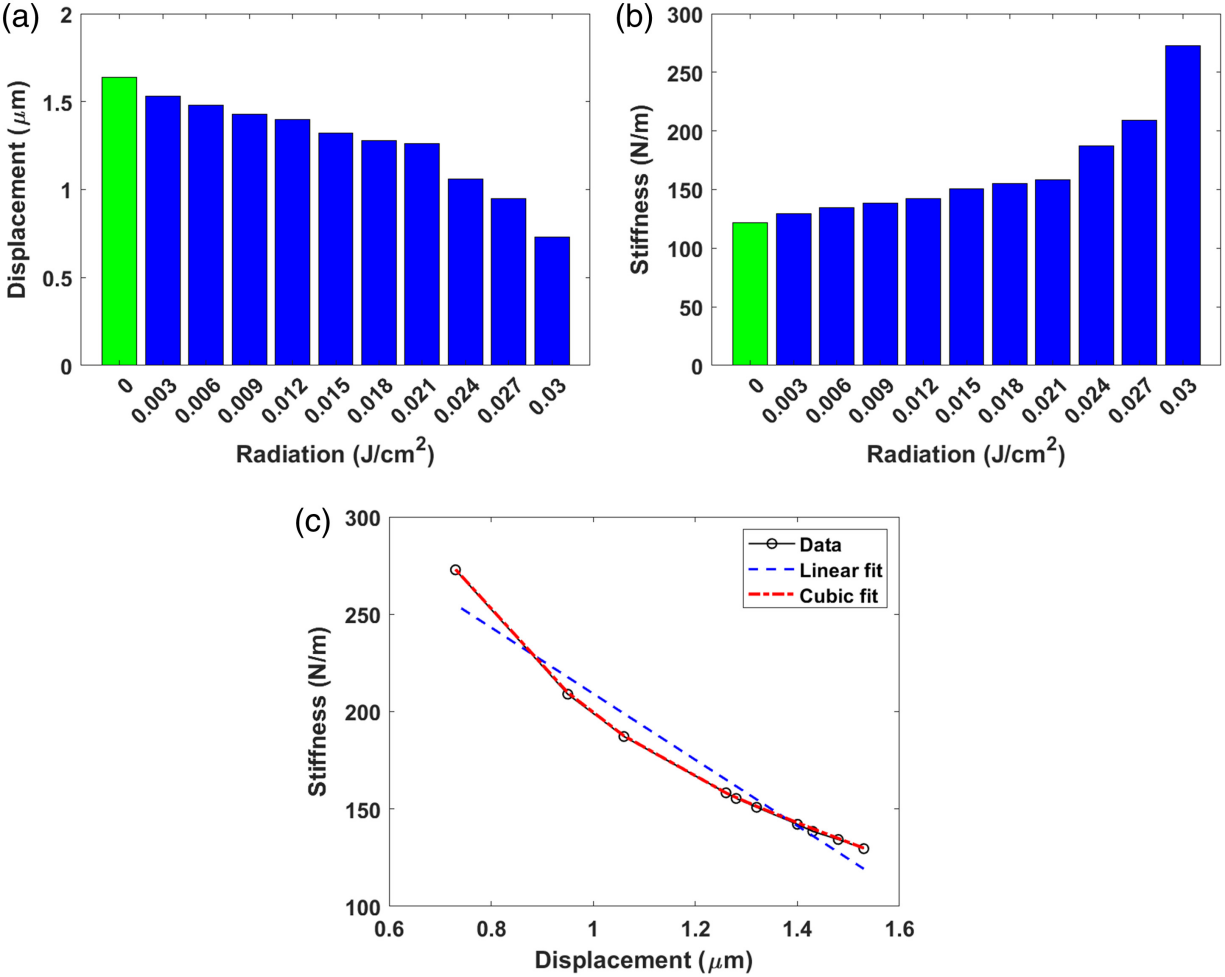
(a) Displacement and (b) stiffness plots showing as a function of radiation dose; (c) plot of stiffness versus displacement by applying a linear and cubic fit to the data. The plots show the changes produced by UV radiation in the M1 test.

To detect alteration of the skin tissue before and after radiation, the displacement and stiffness graphs are presented in Figs. 12(a) and 12(b), respectively, and the relationship between displacement and stiffness is presented in Fig. 12(c). The graphs shown used the data in Table 1 (M1): the UV radiation (first column) and Displacements/stiffness (second column). The first plotted value of Figs 12(a) and 12(b) refers to the non-irradiated sample; the remaining values of the data correspond to the radiated sample. The resulting graphs show that the HST is less elastic because the displacement decreases as the UV exposure levels are increased; the opposite is true for the stiffness, as compared with the values found for the control (non-irradiated) sample. A negative correlation is observed between the amount of UV energy applied and the changes in displacements, whereas a positive correlation is seen between stiffness and UV dose; those data indicate that UV light induces skin stiffening. Fig. 12(c) reveals that the behavior is quasi-linear, i.e., it demonstrates that the skin tissue shows increased stiffness, which is an indicator of loss of elasticity when exposed to different doses of UV radiation. For the irradiated sample, the decrease in displacement is not constant, as shown in the black plot (using the symbol 
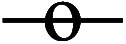
). According to Fig. 12(c), it can be seen that the relationship between displacements and stiffness fits the cubic model, i.e., the fitting curve passes through all points of the original data, whereas the linear fit is poor. It is important to mention that the displacement and stiffness graphs for different tests and measurements (M2 and M3) shown in Table 1 are quite similar to Fig. 12.

The data on displacement and stiffness depicted in the graphs suggest a change in the structure of collagen and elastin fibers within the dermis, which can be attributed to the impact of UV radiation on these important structures. Further, the data indicate that accumulated UV radiation exposure leads to denser structures which causes deterioration or aging of the skin, altering its viscoelastic properties, potentially resulting in dehydration and increased rigidity of the skin.

The values of the mean, variance, and standard deviation of the displacements and stiffness were calculated for samples with UV irradiation using the data in Table 1 (M1), resulting in 1.24  μm/167.816  N/m, $0.065\;\mu m^{2}/1969.3\;N^{2}/m^{2}$, and 0.2557  μm/44.377  N/m, respectively. The percentage deviation was calculated for the displacement and stiffness, resulting in 20.62% and 26.44%, respectively. The percentage deviation also was calculated for the radiated sample in reference to the control sample, resulting in 15.52% for the displacement and 36.77% for stiffness.

The above analysis shows that the stiffness parameter is crucial because it provides useful information; for example, its quantification, related to a high value, might be associated with some diseases, and this may be beneficial in the diagnosis of related pathologies of the skin. In addition, the graphs shown in Fig. 12 serve as calibration charts and thus can be used to predict, from the optical data, displacement, and stiffness as a function of UV dose. Furthermore, some critical changes in skin stiffness may be helpful in generating precise and controlled treatments to achieve better skin care.

## 4 Conclusions

Our research on the effects of UV irradiation on human skin has provided valuable information on the responses of this tissue to UV rays and the results showed that UV radiation caused alterations in the skin. With the aid of the optical non-invasive DHI technique offers a whole field of view assessment of the UV effects on the HST without touching the skin. With the assist of DHI, it is possible to retrieve the skin phase map, and from it, valuable data of the skin surface displacement, and hence its stiffness, can be found. The application of increasing doses of UV radiation at a specific and fixed time resulted in a decrease in displacements and elasticity observed in the 3D displacement maps. The fact that these alterations are not readily visible to the naked eye but were detected with DHI highlights the relevance of the technique, which provides measurements on a micrometer scale in a safe manner.

A synchronization process was necessary to evaluate the repeatability of the measurements and the images captured. For this purpose, a known study sample was first used, a circular aluminum plate, to verify that the optical configuration and methodology operated correctly, then a skin phantom was utilized for the simulation of HST.

The graphical representation of displacement and stiffness data indicates a structural alteration in the collagen and elastin fibers within the dermis, likely due to the effects of UV radiation on these critical components. Prolonged UV exposure tends to densify these fibers, contributing to skin deterioration or aging by changing its viscoelastic characteristics, potentially leading to dehydration and greater stiffness of the skin.

Potential benefits may derive from the use of DHI, acting as a safeguard for the skin exposed a UV radiation from the sun or artificial sources, and the method can be used in the quality control of artificial skin tissues to be implanted.

The research presented herewith is expected to contribute to a better understanding of UV affectation, and it holds significance in the field of biomedical optics, skin care solutions, and dermatology, as well as in other medical areas, and even mechanical ones because mechanical parameters can be obtained with the results achieved.

## Data Availability

The original contributions presented in the study are included in the article, and further inquiries can be directed to the corresponding author.

## References

[1] Skotarczak K. et al., “Photoprotection: facts and controversies,” Eur. Rev. Med. Pharmacol. Sci. 19, 98–112 (2015). 25635982

[r2] Chinnasamy S. Ramachandran M. Sivaji C., “A study on ultraviolet radiation and its effects,” REST J. Adv. Mech. Eng. 1(2), 1–9 (2022).

[3] Dupont E. Gomez J. Bilodeau D., “Beyond UV radiation: a skin under challenge,” Int. J. Cosmet. Sci. 35(3), 224–232 (2013). IJCMDW 0142-5463 10.1111/ics.12036 23406155

[4] Lipsky Z. W. German G. K., “Ultraviolet light degrades the mechanical and structural properties of human stratum corneum,” J. Mech. Behav. Biomed. Mater. 100, 103391 (2019). 10.1016/j.jmbbm.2019.103391 31419748

[5] Juzeniene A. Moan J., “Beneficial effects of UV radiation other than via vitamin D production,” Dermato-Endocrinol. 4(2), 109–117 (2012). 10.4161/derm.20013 PMC3427189 22928066

[6] Trummer C. et al., “Beneficial effects of UV-radiation: vitamin D and beyond,” Int. J. Environ. Res. Public Health 13, 1028 (2016). 10.3390/ijerph13101028 27775585 PMC5086767

[7] “Ultraviolet radiation,” https://www.who.int/teams/environment-climate-change-and-health/radiation-and-health/non-ionizing/ultraviolet-radiation.

[r8] Krause R. et al., “UV radiation and cancer prevention: what is the evidence?” Anticancer Res. 26(4A), 2723–2727 (2006). ANTRD4 0250-7005 16886683

[r9] Narayanan D. L. Saladi R. N. Fox J. L., “Ultraviolet radiation and skin cancer,” Int. J. Dermatol. 49(9), 978–986 (2010). IJDEBB 1365-4362 10.1111/j.1365-4632.2010.04474.x 20883261

[r10] Modenese A. et al., “Occupational exposure to solar radiation and the eye: a call to implement health surveillance of outdoor workers,” Med. Lav 114(4), 1–11 (2023). 10.23749/mdl.v114i4.14657 PMC10415847 37534422

[r11] Rubeshkumar P. C. et al., “Association between exposure to artificial sources of ultraviolet radiation and ocular diseases: a systematic review protocol,” JBI Evid. Synth. 18(8), 1766–1773 (2020). 10.11124/JBISRIR-D-19-00206 32898369

[12] Behar-Cohen F. et al., “Ultraviolet damage to the eye revisited: eye-sun protection factor (e-sPF^®^), a new ultraviolet protection label for eyewear,” Clin. Ophthalmol. 8, 87–104 (2013). 10.2147/OPTH.S46189 24379652 PMC3872277

[13] Merin K. A. Shaji M. Kameswaran R., “A review on sun exposure and skin diseases,” Indian J. Dermatol. 67, 625 (2022). 10.4103/ijd.ijd_1092_20 PMC9971785 36865856

[14] Garra B. S., “Elastography: history, principles, and technique comparison,” Abdom. Imaging 40, 680–697 (2015). ABIMEL 1432-0509 10.1007/s00261-014-0305-8 25637125

[r15] Singh M. S. Thomas A., “Photoacoustic elastography imaging: a review,” J. Biomed. Opt. 24(4), 040902 (2019). JBOPFO 1083-3668 10.1117/1.JBO.24.4.040902 31041859 PMC6990059

[16] Gubarkova E. V. et al., “Comparison of strain ultrasound elastography with compression optical coherence elastography for breast cancer characterization,” in Int. Conf. Laser Opt. (ILCO), Saint Petersburg, Russian Federation, pp. 1–1 (2022). 10.1109/ICLO54117.2022.9840019

[17] Mendoza-Santoyo F. et al., Full Field Optical Metrology and Applications, IOP Publishing (2022).

[18] Flores Moreno J. M. et al., “DHI contemporary methodologies: a review and frontiers,” Opt. Lasers Eng. 135, 106184 (2020). 10.1016/j.optlaseng.2020.106184

[19] Kumar R. Dwivedi G., “Emerging scientific and industrial applications of digital holography: an overview,” Eng. Res. Express 5, 032005 (2023). 10.1088/2631-8695/acf97e

[r20] Hernández-Montes M. S. et al., “Digital holographic interferometry applied to the study of tympanic membrane displacements,” Opt. Lasers Eng. 49(6), 698–702 (2011). 10.1016/j.optlaseng.2010.12.016

[r21] Salbut L. Krezel J., “Digital holographic interferometer for quasistatic and vibrating micro-objects analysis,” Proc. SPIE 5775, 335–342 (2005). PSISDG 0277-786X 10.1117/12.610683

[r22] Budini N. Mulone C. Vincitorio F. M., “Dynamic analysis of an oscillating membrane by digital holographic interferometry,” Proc. Mater. Sci. 9, 79–86 (2015). JMRPA9 10.1016/j.mspro.2015.04.010

[23] Yang L. et al., “Review of electronic speckle pattern interferometry (ESPI) for three dimensional displacement measurement,” Chin. J. Mech. Eng. 27, 1–13 (2014). 10.3901/CJME.2014.01.001

[24] Creath K. Slettemoen G. Å., “Vibration-observation techniques for digital speckle-pattern interferometry,” J. Opt. Soc. Amer. A 2(10), 1629–1636 (1985). JOAOD6 0740-3232 10.1364/JOSAA.2.001629

[25] Ebrahimi A. P., “Mechanical properties of normal and diseased cerebrovascular system,” J. Vasc. Interv. Neurol. 2(2), 155–162 (2009). 22518247 PMC3317338

[26] Singh G. Chanda A., “Mechanical properties of whole-body soft human tissues: a review,” Biomed. Mater. 16(6), 062004 (2021). 10.1088/1748-605X/ac2b7a 34587593

[27] Kemper B. et al., “Endoscopic double-pulse electronic-speckle-pattern interferometer for technical and medical intracavity inspection,” Appl. Opt. 39(22), 3899–3905 (2000). APOPAI 0003-6935 10.1364/AO.39.003899 18349967

[28] Karuppanan U. Unni S. N. Angarai G. R., “Quantitative assessment of soft tissue deformation using digital speckle pattern interferometry: studies on phantom breast models,” J. Med. Imaging 4(1), 016001 (2017). JMEIET 0920-5497 10.1117/1.JMI.4.1.016001 PMC5285730 28180134

[29] Acosta L. et al., “Study of skin rigidity variations due to UV radiation using digital holographic interferometry,” Opt. Lasers Eng. 126, 105909 (2020). 10.1016/j.optlaseng.2019.105909

[30] Takeda M. Ina H. Kobayashi S., “Fourier-transform method of fringe-pattern analysis for computer-based topography and interferometry,” J. Opt. Soc. Amer. 72(1), 156–160 (1982). 10.1364/JOSA.72.000156

[31] Kreis T., Handbook of Holographic Interferometry: Optical and Digital Methods, Wiley (2004).

[32] Ostrovsky Y. I. Shchepinov V. P. Yakovlev V. V., “Holographic interferometry,” in Holographic Interferometry in Experimental Mechanics, Springer Series in Optical Sciences, Vol. 60, Springer, Berlin, Heidelberg (1991).

[r33] Varol R. et al., “Acousto-holographic reconstruction of whole-cell stiffness maps,” Nat. Commun. 13, 7351 (2022). NCAOBW 2041-1723 10.1038/s41467-022-35075-x 36446776 PMC9709086

[r34] Muñoz Solís S. Hernández-Montes M. S. Mendoza Santoyo F., “Measurement of Young’s modulus in an elastic material using 3D digital holographic interferometry,” Appl. Opt. 50, 3383–3388 (2011). APOPAI 0003-6935 10.1364/AO.50.003383 21743544

[r35] Graham H. K. et al., “How stiff is skin?,” Exp. Dermatol. 28(Suppl 1), 4–9 (2019). EXDEEY 0906-6705 10.1111/exd.13826 30698873

[36] Roig-Rosello E. et al., “Dermal stiffness governs the topography of the epidermis and the underlying basement membrane in young and old human skin,” Aging Cell 23(4), e14096 (2024). 10.1111/acel.14096 38475908 PMC11019137

[37] Ballou G., Handbook for Sound Engineers, 4th ed., pp. 24–26, Focal Press (2008).

[r38] Pierce A. D., Acoustics: an Introduction to Its Physical Principles and Applications, 3rd ed., p. 69, Springer (2019).

[39] Schomer P. D. Swenson G. W., “Electroacoustics,” in Reference Data for Engineers, Middleton W. M. Van Valkenburg M. E., Eds., pp. 40-1–40-28, Elsevier (2002).

[40] Madrazo de la Rosa K. et al., “Study and characterization of global solar radiation and incident UV in the city of Leon, Guanajuato, Mexico,” Nova Scientia 15(30), 1–12 (2023). 10.21640/ns.v15i30.3093

